# Supplementary material for: Causal interplay between lactose intolerance and gut microbiota: a combined bidirectional Mendelian randomization and in vivo validation study
Source: Front Nutr. 2026 Jun 1;13:1803337. doi: 10.3389/fnut.2026.1803337 (PMC13265576; doi:10.3389/fnut.2026.1803337)
Supplement: Supplementary file 1 [file Data_Sheet_1.zip › supplementary materials/Forward/leave one out cross validation/ebi-a-GCST90027690.finngen_R12_E4_LACTONAS.pdf]

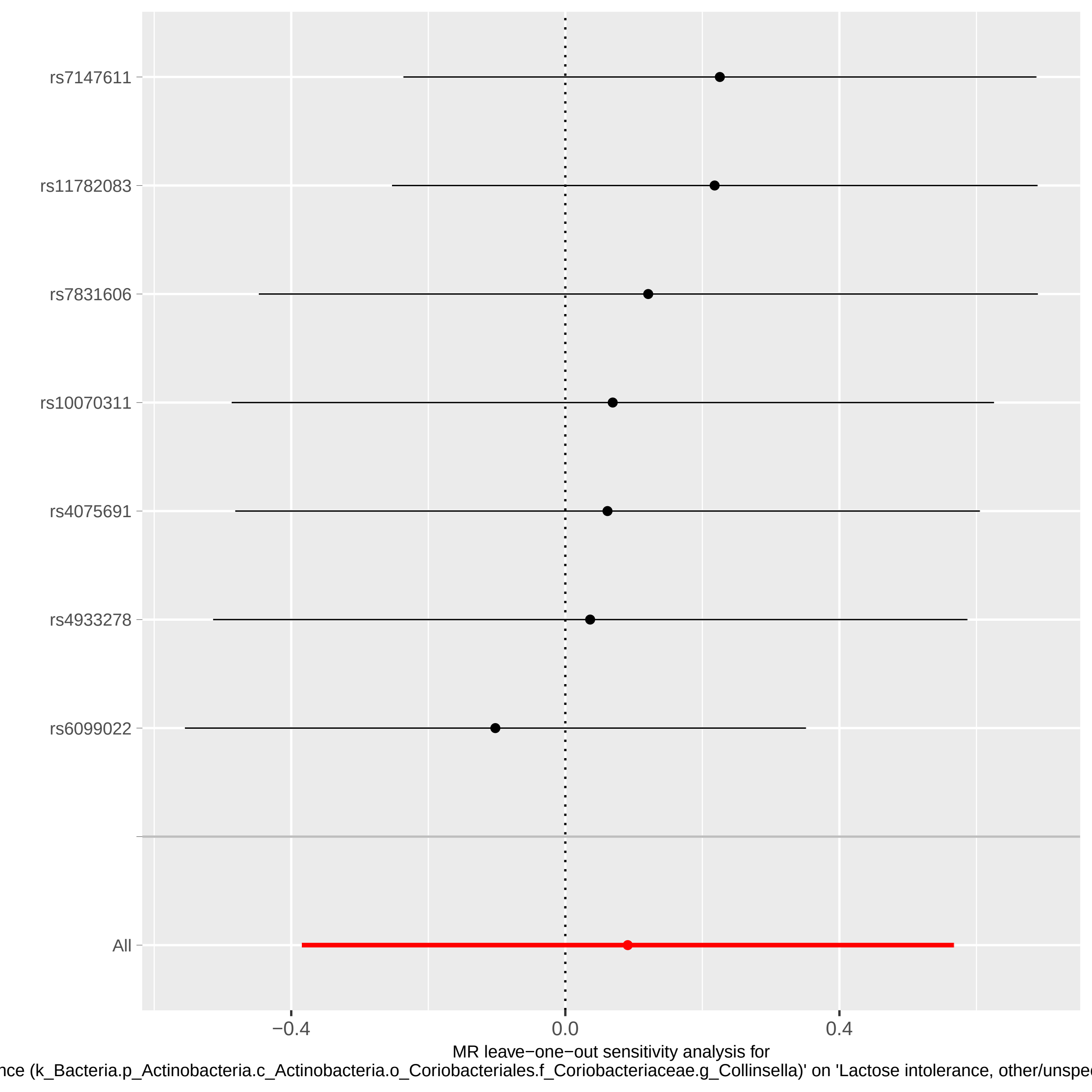
rs7147611
rs11782083
rs7831606
rs10070311
rs4075691
rs4933278
rs6099022
All
−0.4
0.0
0.4
MR leave−one−out sensitivity analysis for
nce (k_Bacteria.p_Actinobacteria.c_Actinobacteria.o_Coriobacteriales.f_Coriobacteriaceae.g_Collinsella)' on 'Lactose intolerance, other/unspe
